# A saliency-specific and dimension-independent mechanism of distractor suppression

**DOI:** 10.3758/s13414-020-02142-8

**Published:** 2020-10-06

**Authors:** Dongyu Gong, Jan Theeuwes

**Affiliations:** 1 Department of Psychology, Tsinghua University, Haidian District, Beijing, 100084 China; 2 Department of Experimental and Applied Psychology, Vrije Universiteit Amsterdam, Amsterdam, Netherlands

**Keywords:** Suppression, Saliency, Attentional capture, Visual attention

## Abstract

During everyday tasks, salient distractors may capture our attention. Recently, it was shown that through implicit learning, capture by a salient distractor is reduced by suppressing the location where a distractor is likely to appear. In the current study, we presented distractors of different saliency levels at the same specific location, asking the question whether there is always one suppression level for a particular location or whether, for one location, suppression depends on the actual saliency of the distractor appearing at that location. In three experiments, we demonstrate a saliency-specific mechanism of distractor suppression, which can be flexibly modulated by the overall probability of encountering distractors of different saliency levels to optimize behavior in a specific environment. The results also suggest that this mechanism has dimension-independent aspects, given that the saliency-specific suppression pattern is unaffected when saliency signals of distractors are generated by different dimensions. It is argued that suppression is saliency-dependent, implying that suppression is modulated on a trial-by-trial basis contingent on the saliency of the actual distractor presented.

**Electronic supplementary material:**

The online version of this article (10.3758/s13414-020-02142-8) contains supplementary material, which is available to authorized users.

## Public significance statement

It is important to be able to avoid distraction from salient objects. Previous studies have shown that we can extract spatial and feature regularities from the visual environment, which in turn leads to optimized attentional control. In the current study, we show that suppression at a particular location can be selectively adjusted to the saliency level of the distractor presented at that location. In other words, the amount of suppression at a particular location is contingent on the saliency of the distractor appearing at that location. It is argued in favor of saliency-dependent suppression that modulates suppression on a trial-by-trial basis, contingent on the saliency of the actual distractor presented.

## Introduction

In everyday life, at any moment in time, our visual system receives massive amounts of information (K. Koch et al., 2006). Due to the limited amount of cognitive resources available (Broadbent, 1958; Lennie, 2003), we must select information that is relevant to us while ignoring irrelevant stimuli that may distract us. It is generally agreed that attentional deployment can be biased by both physical saliency of the object (i.e., bottom-up, stimulus-driven selection) and current goals in the task (i.e., top-down, goal-oriented selection; Corbetta & Shulman, 2002; Theeuwes, 2010). Under this framework, many models of attentional control have proposed that the early features of the visual scene will be computed hierarchically to generate conspicuity maps for different feature dimensions, and then the conspicuity maps are combined into a unique saliency map, which encodes for saliency independent of feature dimensions in a topographical fashion (Itti & Koch, 2000; C. Koch & Ullman, 1987). At the same time, during most stages of feature computing, top-down signals from higher-level brain areas can bias the cortical representations of certain feature values that are related to current selection goals, thus modulating the weights of corresponding feature channels in the saliency map (see Itti & Koch, 2001, for a review of traditional saliency models). The saliency map then determines where we attend, with the most salient location prioritized even when the item there is a distractor. The phenomenon that salient-but-irrelevant distractors can capture our attention automatically is known as *attentional capture* (Hickey, McDonald, & Theeuwes, 2006; Theeuwes, 1992), although some researchers argue that attention is only captured by features that match current selection goals (Folk & Remington, 1998).

Recently, the role of selection history in shaping saliency has been dissociated from goal-oriented selection (Awh, Belopolsky, & Theeuwes, 2012). Some researchers have claimed that items which have been previously attended (Hillstrom, 2000; Theeuwes & Van der Burg, 2011), or items associated with reward value (Failing & Theeuwes, 2018; Hickey, Chelazzi, & Theeuwes, 2010), will elicit selection biases that cannot be explained by physical saliency of objects or current selection goals.

Importantly, research on the consequences of selection history, particularly regularities that we experienced, has demonstrated not only lingering selection biases toward specific locations when the target bears certain regularities (Chun & Jiang, 1999), but also biases away from or suppression of distractors that were consistently cued or consistently displayed at certain locations (Goschy, Bakos, Müller, & Zehetleitner, 2014; Leber, Gwinn, Hong, & O’Toole, 2016; Wang & Theeuwes, 2018b). For example, Wang and Theeuwes (2018b) used a variant of the additional singleton paradigm (Theeuwes, 1991, 1992) where participants were to search in a circular array for a shape singleton while ignoring an irrelevant color singleton. Crucially, one of the locations had a higher probability of containing a distractor than the other locations. Results showed that when a distractor was presented at this high-probability location (HPL), attentional capture by the distractor was reduced relative to when it appeared at the low-probability locations (LPLs). Moreover, on distractor-absent trials, when the target appeared at the HPL, search performance was less efficient relative to when it appeared at any of the other locations. These results were interpreted as evidence that through statistical learning of the spatial regularities, within the saliency map relative to the LPLs, the HPL was suppressed such that this location competed less for attention than other locations. More recently, it was claimed that attentional suppression can also be induced by statistical learning of feature regularities (Failing, Feldmann-Wustefeld, Wang, Olivers, & Theeuwes, 2019; Stilwell, Bahle, & Vecera, 2019). For example, Failing et al. (2019) presented one distractor feature (e.g., a red distractor) more often at one location and another distractor feature (e.g., a green distractor) more often at the other location. They found that suppression was more efficient when a distractor was presented at the HPL that matched its feature, relative to when it appeared at the HPL of the other distractor feature.

The interaction of spatial and feature-specific processing in attentional suppression can be considered in terms of traditional saliency models and neurobiological substrates of selective visual attention. From the perspective of traditional saliency models, suppressing a feature value at a specific location will establish a history-driven suppression for this feature value at that specific location in its feature map. Suppression from the feature maps then may spread to their dimension-specific conspicuity maps (Failing et al., 2019). The saliency map (which combines conspicuity maps) is modulated correspondingly such that selective attention functions to avoid distraction in both spatial and feature-specific fashion. On the other hand, according to the biased competition theory of visual attention (Deco & Zihl, 2001; Desimone & Duncan, 1995) in neurobiology, attending to a stimulus will enhance its neural representation compared with other stimuli present in the visual field. This suggests that attentional capture by a distractor at a particular location or with a particular feature will bias the neural representation towards that location or feature, such that neurons representing a highly probable location or feature are more often activated. This might lead to the gradual decrease of responsiveness of those neurons due to neural adaptation (Carandini, 2000; Clifford et al., 2007; Shapley & Enroth-Cugell, 1984) and finally cause spatial and feature-specific suppression.

Recently, Failing and Theeuwes (2020) used another variant of the additional singleton paradigm with two HPLs, one for low-saliency and one for high-saliency distractors, respectively. They claimed that the more salient a distractor, the more suppression was applied to the HPL of that distractor. This means that observers can learn to suppress selectively different locations that contain distractors that have either high or low saliency. The results suggest that the specific suppression is bound to the saliency of the distractor appearing more often at that specific location. However, what is not clear from this study is whether there is always one suppression level for a particular location or whether, even for one location, suppression depends on the actual saliency of the distractor appearing at that location. In other words, is it possible to have selective suppression of different saliency values even when these distractors within a block randomly appear at one specific HPL? The question is then when distractors of different saliency levels have the same HPL, will all the distractors be suppressed with the same (average) magnitude, or each distractor will be suppressed in accordance with their own saliency, respectively?

The question we address here has important implications for theories of attentional selection. Imagine distractors defined within the same feature dimension but of different saliency levels share the same HPL. Due to statistical learning, biasing presentation of distractors to the HPL will result in a history-driven suppression at that location in the conspicuity map of that dimension. The question is, when distractors of different saliency levels are displayed randomly within a block of trials, will they elicit an “across-trial” average magnitude of suppression within the saliency map, or will the suppression be adaptive to the trial-to-trial saliency of a distractor presented at the HPL on a given trial?

In an effort to distinguish among multiple potential mechanisms of distractor suppression, Gaspelin and Luck (2018) claimed that first-order feature information is required to suppress distractors, so that suppression is achieved only when there is foreknowledge of the upcoming distractor’s feature value (e.g., red, vertical), a finding which also has been observed in earlier studies (Graves & Egeth, 2016; Kerzel & Barras, 2016). However, there is also evidence in favor of second-order singleton suppression (Sauter,Liesefeld, & Müller, 2019; Won, Kosoyan, & Geng, 2019), which implies local feature discontinuities on a specific feature dimension can be suppressed even when foreknowledge of the upcoming distractor’s feature value is not available. Besides first-order and second-order feature suppression models, another hypothesis proposes a global saliency suppression model, according to which the visual system can suppress a salient distractor irrespective of feature dimensions, but so far there is little, if any, direct evidence for this model (Gaspelin & Luck, 2018). In this sense, the current study that examines whether there is saliency-specific and dimension-independent distractor suppression due to implicitly learned regularities will shed light on the mechanisms of distractor suppression that are not yet fully understood.

The current study used the same HPL for different distractors each having different saliency levels. Participants were to search for a singleton shape target while ignoring a singleton distractor. In Experiment 1, circles that varied in diameter were used as distractors generating low, medium, and high saliency levels. Each of these distractors having a different level of saliency was equally likely to appear more often at one specific location (HPL) than at all other locations (LPLs). We hypothesized that if there is saliency-specific suppression, the amount of suppression should be contingent on the saliency of the distractor presented at any given trial. That is, on any given trial, a high, medium, or low-saliency distractor should receive on that specific trial a high, medium, or low amount of suppression at the HPL relative to when they appear at the LPLs, respectively. Alternatively, it is possible that there is an “across-trial” average suppression that is applied to all distractors presented at the HPL regardless of their saliency.

The current study is also novel in that it used distractors defined along a dimension that has seldom been explored in attentional capture research. While most research has explored the effects of singletons defined by color, shape, and orientation differences, it is known that size is another dimension that affects the deployment of attention (Wolfe, 2016, 2017; Wolfe & Horowitz, 2004). If size can guide attention towards certain items, it is plausible that it can be used to suppress irrelevant distractors. However, to our knowledge, there are no previous studies that have examined suppression of distractors that varied in size. Therefore, the current study should also have the merit of testing the generality of previous findings.

**Fig. 1:**
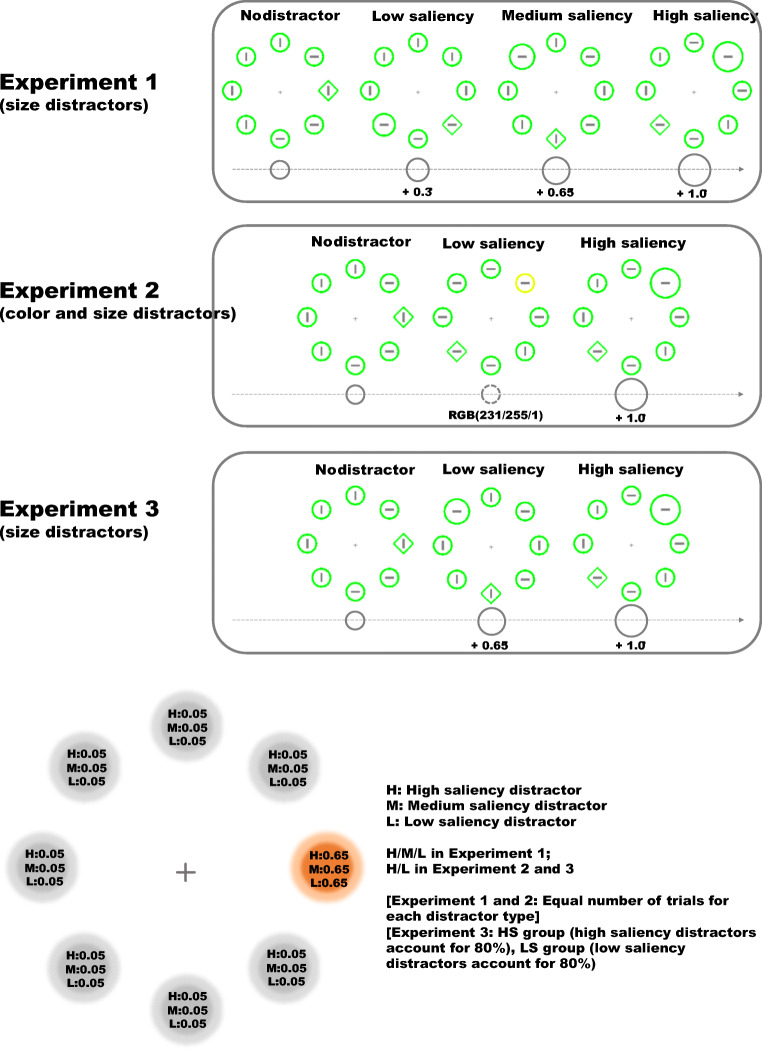
Upper panel: Illustrations of search displays in Experiments 1, 2, and 3 showing possible distractor types and locations. The actual displays used in the experiments were presented on a uniform black background. Bottom panel: Probability distribution of appearing at different locations for different distractor types in Experiments 1, 2, and 3

In Experiment 1, we assessed different saliency levels of a distractor within the same dimension. In Experiment 2, we tested whether the putative saliency-specific suppression is confined to a single feature dimension (i.e., within size dimension) or whether this kind of suppression is independent of feature dimensions (i.e., consistent across size and color dimensions). To this end, high-saliency distractors defined on size dimension and low-saliency distractors defined on color dimension were presented and shared the same HPL. In Experiment 3, we further investigated whether the frequency of displaying distractors of different saliency levels across trials would reshape the putative saliency-specific suppression. High-saliency and low-saliency distractors were presented at the same HPL, but for different groups of participants the frequency of encountering high-saliency and low-saliency distractors was manipulated. For half of participants, 80% of distractor-present trials contained a high-saliency distractor, while 20% contained a low-saliency one, and vice versa for the other half of participants (see Fig. 1 for illustrations of Experiments 1, 2, and 3). We expect that participants can learn the regularities regarding the occurrence of distractor saliency across trials and will bias their attention accordingly. In all experiments, we planned comparisons of participants’ performance in different distractor saliency conditions (including a no-distractor condition) in advance of the data collection to confirm whether the distractors we defined did interfere with target search and, crucially, whether the interference varied with the saliency level of the distractor.

## Experiment 1

### Method

#### Participants

A total of 32 students (10 males, 22 females, *M*_age_ = 20.94 years, *SD* = 3.67) from Vrije Universiteit Amsterdam, with reported normal or corrected-to-normal visual acuity, were recruited and received course credits or monetary payment for their participation. The experiment was approved by the Ethical Review Committee of the Faculty of Behavioral and Movement Sciences of Vrije Universiteit Amsterdam, and all participants signed the informed consent before any experimental procedure began.

#### Apparatus and stimuli

An HP Compaq Pro 6300 SFF computer with a 22-inch liquid crystal display (LCD) color monitor (1,680 × 1,050-pixel resolution, 120-Hz refresh rate) was used in the experiment. The experiment was programmed in MATLAB (The MathWorks, Inc., Natick, MA) using the Psychophysics Toolbox (Brainard, 1997) and presented on a uniform black background (RGB: 5/5/5, luminance: 0.50 cd/m^2^) at a distance of 72 cm. The search display contained seven green circles and one green diamond (RGB: 0/255/0, luminance: 46 cd/m^2^), which was displayed at equal distance on an imaginary circle centered at the fixation, with an eccentricity of 4.35 degrees of visual angle (abbreviated as d.v.a. hereafter). On distractor-absent trials, all the stimuli had the same size (the circles had a diameter of 1.8 d.v.a. and the diamond subtended 1.8 d.v.a. × 1.8 d.v.a.). On distractor-present trials, one of the circles deviated in size from all other circles, with an increment of 0.3, 0.65 and 1.0 d.v.a. in diameter for low, medium, and high-saliency distractors correspondingly. In each display, there was a gray (RGB: 127/127/127, luminance: 18 cd/m^2^) line segment (with a length of 0.8 d.v.a. and a thickness of 0.08 d.v.a.) within all the shapes, which was either horizontal or vertical. The intertrial interval was set at 500 ms.

#### Procedure and design

Each trial began with a fixation display which lasted randomly between 700 ms to 1,000 ms. Then, the search display was presented for 1,500 ms or until response. Participants had to search for the uniquely shaped singleton (a diamond presented among seven circles) and indicate the orientation of the line segment inside the target on a keyboard (“S” for horizontal and “K” for vertical). An incorrect response or failing to respond within the time window would trigger a warning sound in the earphone. All the instructions were presented on the screen. All participants were required to reach an accuracy of 85% or higher in a practice session of 20 trials, and were asked to repeat the practice if they did not reach the requirement.

After the practice session, each participant performed 12 experimental blocks of 180 trials each. One-sixth of the trials were distractor-absent trials, while on the remaining trials, a low, medium, or high-saliency size distractor was presented. Crucially, each of these three types of distractors was more likely (65% probability) to appear at one of the eight locations in the search display, whereas the three types of distractors appeared equally often across all trials. For each participant, this location (the HPL) was the same for the low, medium, and high-saliency distractors, but the location itself was counterbalanced across participants. When a distractor did not appear at the HPL, it was equally likely to appear at any of the remaining locations (the LPLs). The target could appear equally often at any location on distractor-absent trials. On distractor-present trials, the target’s position, which should be one of the seven locations that was not currently occupied by the distractor, was determined randomly with equal probability at each location. The orientation of the line segment contained within each shape was also randomly set in all trials.

After the experiment, participants were required to fill in an implicit learning questionnaire with two forced-choice questions. For the first question, they were asked whether they noticed any regularities regarding the locations where the distractors were presented. For the second question, they were explicitly informed that there was one location that had a larger probability to display a distractor, and were asked to indicate that location. Note that it was not possible to change the response to the first question after seeing the second question (which revealed the answer to the first question), because they were presented on the computer screen in a fixed sequence.

### Results

Only trials with a correct response were used in the analyses. One participant had an abnormally low accuracy (with an error rate over 70% and higher than 2.5 standard deviations from the group mean) and was excluded from the analyses, resulting in a valid sample size of 31. For the remaining participants, trials on which the response times (RTs) were faster than 200 ms (1.7%) were excluded from the analyses.

#### Attentional capture

**Fig. 2:**
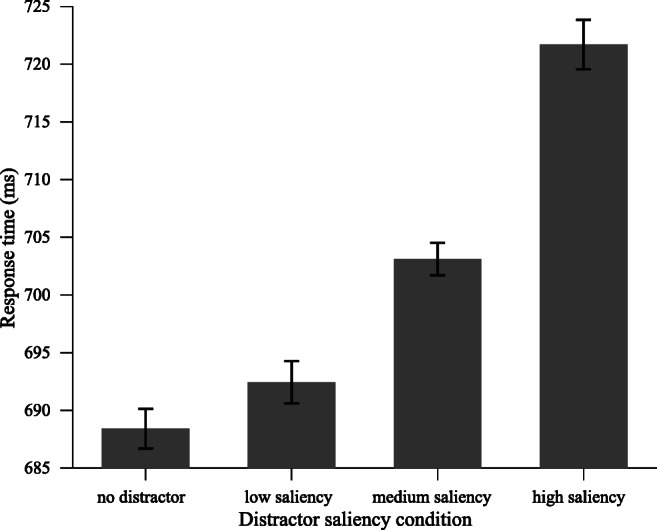
Experiment 1: Mean RTs for different distractor saliency conditions. Error bars here and in all the following figures, denote ±1 the standard error of the mean

To assess the impact of our saliency manipulation on attentional capture, we performed a one-way repeated-measures analysis of variance (ANOVA) on mean RTs and mean error rates with distractor presence (absent vs. low-saliency distractor vs. medium-saliency distractor vs. high-saliency distractor) as a factor. As shown in Fig. 2, the effect on RTs was reliable, *F*(3, 90) = 51.48, *p* < .001, ${\eta}_p^2$= .63. Planned comparisons revealed

that low, medium, and high-saliency distractors all interfered with target search (absent vs. high-saliency distractor: *M* = 688 ms ± *SD* = 80 vs. 722 ms ± 85), *t*(30) = 9.37, *p* < .001, unbiased Cohen’s *d* (also known as Hedges’s *g*; see Cumming, 2012) = 0.40; (absent vs. medium-saliency distractor: 688 ms ± 80 vs. 703 ± 82), *t*(30) = 5.29, *p* < .001, *d* = 0.18; (absent vs. low-saliency distractor: 688 ms ± 80 vs. 692 ms ± 78), *t*(30) = 1.93, *p* = .064, marginally significant. Crucially, the high-saliency distractor caused larger interference than the medium-saliency distractor, *t*(30) = 8.42, *p* < .001, *d* = 0.22, and the medium-saliency distractor caused larger interference than the low-saliency distractor, *t*(30) = 3.71, *p* < .001, *d* = 0.13. The results on error rates were congruent with (i.e., the same direction as) those for RTs, *F*(3, 90) = 3.41, *p* = .021, ${\eta}_p^2$ = .10, which showed that the RT differences were not the consequence of a speed–accuracy trade-off. This demonstrates that our saliency manipulation successfully caused attentional capture, and the amount of capture did increase with the saliency of the distractor.

#### Saliency-specific suppression

**Fig. 3:**
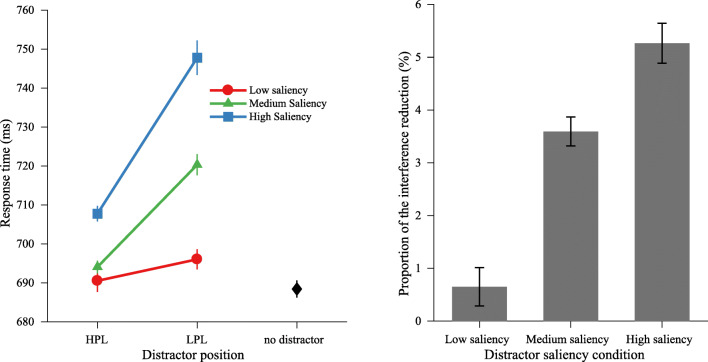
Experiment 1. Left panel: Mean RTs by distractor saliency over distractor position condition. Right panel: The proportion of reduction in interference (from the LPL to the HPL) for different distractor saliency conditions

To examine the hypothesis of a saliency-specific suppression—that is, to see whether the amount of suppression differed between the distractors having different saliency levels—we submitted RT data to an ANOVA with the factors distractor saliency (low vs. medium vs. high) and distractor position (HPL vs. LPL). As shown in Fig. 3 (left panel), the results showed a main effect of distractor saliency, *F*(2, 60) = 55.79, *p* < .001, ${\eta}_p^2$= .65, and a main effect of distractor position, *F*(1, 30) = 53.60, *p* <.001, ${\eta}_p^2$= .64. Crucially, there was significant interaction, *F*(2, 60) = 29.43, *p* < .001, ${\eta}_p^2$= .50, between these two factors. Subsequent comparisons showed that the amount of suppression (RT when the distractor appeared at the HPL minus RT when the distractor appeared at the LPL) increased as the saliency of the distractor varied from low to medium, low to high, and medium to high (low vs. medium: 5.5 ms ± 19.8 vs. 26.3 ms ± 19.6), *t*(30) = 5.42, *p* < .001, *d* = 1.04; (low vs. high: 5.5 ms ± 19.8 vs. 40.0 ms ± 29.3), *t*(30) = 6.42, *p* < .001, *d* = 1.36; (medium vs. high: 26.3 ms ± 19.6 vs. 40.0 ms ± 29.3), *t*(30) = 3.25, *p* = .003, *d* = 0.55. The interference by the low-saliency distractor was significant when it appeared at the LPL, *t*(30) = 2.52, *p* = .017, but was reduced to be statistically undistinguishable from search performance on distractor-absent trials when it appeared at the HPL, *t*(30) = 0.85, *p* = .404, BF_01_ (Bayes factor) = 3.76, which indicates that the data was considerably more consistent with the null hypothesis. However, the interference by the medium saliency distractor when appearing at the HPL was statistically significant from distractor absent trials, *t*(30) = 2.38, *p* = .024. Similar analyses on error rate only showed a main effect of distractor saliency, but no main effect of distractor position or interaction (see Table 1 for mean RTs and error rates).

**Table 1:** Mean response times (RTs) and error rates of all experiments (*M* ± *SD*)

Experiment		Distractor saliency	Distractor position	RT (ms)	Error rate (%)
1		Low	HPL	691 ± 76	8.5 ± 4.6
			LPL	696 ± 84	8.3 ± 4.6
		Medium	HPL	694 ± 81	8.2 ± 4.6
			LPL	720 ± 86	8.6 ± 5.2
		High	HPL	708 ± 83	8.8 ± 5.0
			LPL	748 ± 90	9.7 ± 5.4
		Absent		688 ± 80	8.2 ± 4.6
2		Low	HPL	715 ± 97	9.7 ± 7.1
			LPL	734 ± 92	10.3 ± 7.2
		High	HPL	728 ± 106	10.4 ± 8.2
			LPL	767 ± 98	10.7 ± 8.2
		Absent		705 ± 98	9.5 ± 7.6
3	LS group	Low	HPL	696 ± 79	7.3 ± 4.2
			LPL	715 ± 80	8.0 ± 3.8
		High	HPL	699 ± 77	7.8 ± 4.6
			LPL	745 ± 74	9.2 ± 5.0
		Absent		691 ± 72	7.1 ± 4.8
	HS group	Low	HPL	664 ± 79	5.1 ± 2.3
			LPL	691 ± 87	6.7 ± 2.7
		High	HPL	673 ± 81	5.5 ± 2.7
			LPL	696 ± 84	6.4 ± 3.2
		Absent		669 ± 84	5.6 ± 2.6

However, there is an alternative explanation of our saliency-specific suppression results: It is possible that there is a certain proportion by which the initial interference of any distractor is reduced. This implies that when the distractor has a high saliency level, the initial interference is strong, and the amount of suppression will be large as well. To test this possibility, we performed a one-way repeated measures ANOVA on the proportion of the interference reduction, with distractor saliency (low vs. medium vs. high) as a factor. The proportion of the interference reduction (***P***), as calculated by the formula below, represents the ratio of RT reduction (from the LPL to the HPL) to RT at the LPL:

1 $$ \boldsymbol{P}=\frac{RT\ (LPL)- RT(HPL)\ }{RT(LPL)}. $$

As shown in Fig. 3 (right panel), the results showed a main effect of distractor saliency, *F*(2, 60) = 31.04, *p* < .001, ${\eta}_p^2$ = .51. Planned comparisons revealed that the interference of highly salient distractors was reduced by a larger proportion than lower salient distractors (low vs. medium: 0.7% vs. 3.6%), *t*(30) = 5.65, *p* < .001, *d* = 1.13; (low vs. high: 0.7% vs. 5.3%), *t*(30) = 6.68, *p* < .001, *d* = 1.43; (medium vs. high: 3.6% vs. 5.3%), *t*(30) = 3.02, *p* = .005, *d* = 0.52. These findings suggest that a proportional suppression explanation is less likely. Instead, the findings are consistent with a suppression that is contingent of the actual saliency of the distractor.

Above all, our findings support the notion of a saliency-dependent suppression: When distractors of different saliency levels are presented at the same HPL, the amount of suppression is determined by the saliency of the distractor per se.

#### Awareness assessment

Out of 31 participants, nine indicated on the implicit learning questionnaire that they noticed certain regularities. Of those nine participants, only two correctly identified the HPL in the second question. After excluding these two participants from all analyses, all major findings remain the same (see Supplemental Materials).

### Discussion

In Experiment 1, we found evidence for saliency-specific suppression: when distractors having different saliency levels are presented at the same HPL, the amount of suppression was larger for distractors of a high saliency level relative to those of a low saliency level. Also, the spatial distribution of the suppression effect exhibited a saliency-specific pattern (see Supplemental Materials). As all distractors were presented at the same HPL, the difference in suppression for distractors of different saliency levels cannot be explained by a mechanism that is location-based only (see also Failing et al., 2019, for a similar argument). We argue that in addition to location-based suppression, another mechanism has to be assumed that modulates suppression on a trial-by-trial basis contingent on the saliency of the actual distractor presented. Note that this cannot be based on the “pretrial” expected saliency, as each distractor having a particular saliency level was equally often presented at the HPL in a random order.

## Experiment 2

In Experiment 1 we observed saliency-specific suppression for different distractors defined on size dimension. However, it is also important to assess whether such a mechanism is confined within a single feature dimension or can extend to different feature dimensions.

We addressed this question in Experiment 2 by using a high-saliency size distractor and a low-saliency color distractor. The size distractor and the color distractor were more likely to appear at the same HPL. If saliency-specific suppression is independent of the feature dimension that generates the saliency signal, similar results as in Experiment 1 are expected. That is, more suppression should be applied to the HPL when a high-saliency size distractor appears relative to when a low-saliency color distractor appears. If, however, suppression breaks down because it cannot be set at one location for different feature dimensions, we would expect to find no or an “across-trial” average suppression effect that does not depend on the saliency of the distractor presented at that location.

### Method

Another group of 32 students (12 males, 20 females, *M*_age_ = 20.53 years, *SD* = 1.92) from Vrije Universiteit Amsterdam, with reported normal or corrected-to-normal visual acuity, participated in Experiment 2.

The experimental setup was identical to Experiment 1, with the following exceptions: There were two types of distractors, a low-saliency yellowish color (RGB: 231/255/1, luminance: 71 cd/m^2^) distractor, and a high-saliency size distractor with an increment of 1.0 d.v.a. in diameter (identical to the high-saliency distractor in Experiment 1). All shapes except the low-saliency distractor were green (RGB: 0/255/0, luminance: 46 cd/m^2^) displayed on a uniform black background (RGB: 5/5/5, luminance: 0.50 cd/m^2^). As in Experiment 1, one-sixth of the trials were distractor-absent trials, while on the remaining trials, a low-saliency color distractor or a high-saliency size distractor was present. Crucially, both types of distractors were more likely (65% probability) to appear at one and the same location (the HPL) in the search display, whereas both types of distractors appeared equally often across all trials. The HPL was kept constant among participants but was counterbalanced across participants. When a distractor did not appear at the HPL, it was equally likely to appear at any of the remaining locations. The target appeared equally often at any location on distractor-absent trials. On distractor-present trials, the target’s position was determined randomly with equal probability at one of the seven locations that was not currently occupied by the distractor. The orientation of the line segment contained within each shape were randomly set in all trials. After performing the task, participants finished the same questionnaire as in Experiment 1.

### Results

Trials on which RTs were faster than 200 ms (2.7%) were excluded from the analyses.

#### Attentional capture

To assess whether both types of distractors interfered with target search, and whether the high-saliency size distractor elicited more attentional capture than the low-saliency color distractor, we performed a one-way repeated-measures

**Fig. 4:**
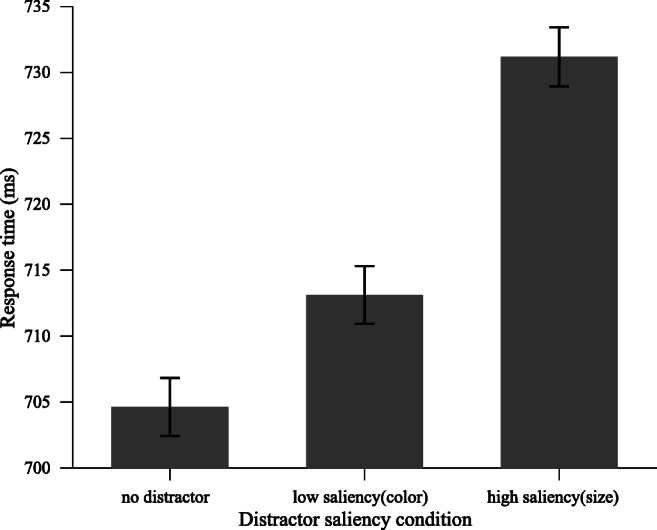
Experiment 2: Mean RTs for different distractor saliency conditions

ANOVA on mean RTs and mean error rates, with distractor presence (absent vs. low-saliency color distractor vs. high-saliency size distractor) as a factor. As shown in Fig. 4, the effect on RTs was significant, *F*(2, 62) = 25.13, *p* < .001, ${\eta}_p^2$= .45. Planned comparisons revealed that both color and size distractors interfered with target search (absent vs. color distractor: 705 ms ± 98 vs. 713 ± 91), *t*(30) = 2.25, *p* = .032, *d* = 0.09; (absent vs. size distractor: 705 ms ± 98 vs. 731 ms ± 99), *t*(31) = 6.87, *p* < .001, *d* = 0.27. Crucially, the high-saliency size distractor caused a larger interference effect than the low-saliency color distractor, *t*(30) = 4.70, *p* < .001, *d* = 0.19, which demonstrates that our saliency manipulation was successful. There were no significant effects on error rates (*p*s > .1).

#### Saliency-specific suppression

**Fig. 5:**
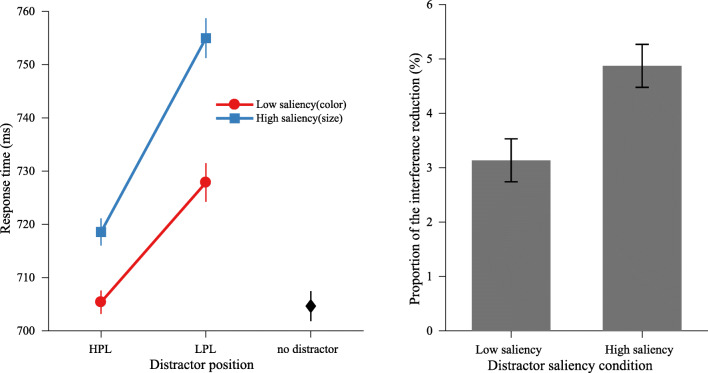
Experiment 2. Left panel: Mean RTs by distractor saliency over distractor position condition. Right panel: The proportion of reduction in interference (from the LPL to the HPL) for different distractor saliency conditions

To examine whether saliency-specific suppression is independent of the feature dimension—that is, whether the interference of the high-saliency size distractor would be reduced by a higher magnitude than the low-saliency 2color distractor, we submitted RT data to an ANOVA with the factors distractor saliency (low vs. high) and distractor position (HPL vs. LPL). As shown in Fig. 5 (left panel), the results showed a main effect of distractor saliency, *F*(1, 31) = 23.35, *p* < .001, ${\eta}_p^2$ = .43, and a main effect of distractor position, *F*(1, 31) = 86.63, *p* <.001, ${\eta}_p^2$= .74. Crucially, the interaction was also reliable, *F*(1, 31) = 5.28, *p* = .028, ${\eta}_p^2$= .15. Subsequent comparisons showed that the magnitude of suppression was higher for the high-saliency size distractor than the low-saliency color distractor (36.4 ms ± 26.1 vs. 22.5 ms ± 23.4), *t*(31) = 2.30, *p* = .028, *d* = 0.55. Notably, when the low-saliency color distractor appeared at the HPL, suppression was so strong that there was no longer a capture effect, as RTs were indistinguishable from distractor-absent trials, *t*(31) = 0.21, *p* = .839, BF_01_ = 5.19, whereas RTs when the high-saliency size distractor appeared at the HPL were still statistically longer than RTs on distractor-absent trials, *t*(31) = 3.79, *p* < .001, *d* = 0.67. Similar analyses on error rate showed no significant effects on distractor saliency, distractor position, or the interaction (see Table 1 for mean RTs and error rates).

Similar to Experiment 1, a *t* test on the proportion of the interference reduction showed that the interference of high-saliency size distractors was reduced by a larger proportion than low-saliency color distractors (low vs. high: 3.1% vs. 4.9%), *t*(31) = 2.20, *p* = .036, *d* = 0.51 (see Fig. 5, right panel), which rules out the possibility that suppression is applied to all distractors by the same proportion of the initial interference.

These results demonstrate that the saliency-specific suppression that we found in Experiment 1 can also occur when the saliency is derived from different feature dimensions. It suggests that the amount of suppression that is applied is determined by the saliency of the distractors and not necessarily by different feature dimensions.

#### Awareness assessment

Out of 32 participants, eleven participants indicated that they noticed certain regularities. Of those eleven participants, only five correctly identified the HPL. After excluding these five participants from all analyses, all major findings remain the same (see Supplemental Materials).

### Discussion

The results of Experiment 2 showed evidence supporting a mechanism of saliency-specific suppression, regardless of whether this saliency is generated by color differences or size differences.

The current results provide direct evidence for global saliency suppression models. Experiment 2 shows that through implicit learning, an object with a strong bottom-up saliency signal can be suppressed, regardless of the feature dimension that generates the signal. As trials on which a low-saliency color distractor or a high-saliency size distractor appeared were fully intermixed, we have to assume that the saliency-based suppression operates on a trial-by-trial basis.

## Experiment 3

In Experiments 1, and 2, we found that the more salient a distractor, the more suppression was applied at the shared HPL. However, it is important to note that in both experiments different types of distractors appeared equally often across all trials. Previous research has shown that the frequency with which a distractor is encountered within a block affects the magnitude of suppression applied (Müller, Geyer, Zehetleitner, & Krummenacher, 2009). Specifically, if a distractor is rarely encountered within a block, it will cause larger interference compared with when it is frequently encountered within a block. However, in the context of the present study, it remains unknown how the frequency of encountering distractors of different saliency levels would affect the saliency-specific suppression effect we found in Experiments 1, and 2.

Experiment 3 was designed to investigate the critical factors underlying saliency-specific suppression. In Experiment 3, we employed two types of size distractors of different saliency levels. Crucially, however, we manipulated the overall probability that a distractor of a particular saliency level would be presented. For one group of participants, the low-saliency distractor appeared more often across all trials (referred as LS group hereafter), while for the other group the high-saliency distractor was more likely to be presented (referred as HS group hereafter).

### Method

A new group of 38 students (16 males, 22 females, *M*_age_ = 20.34 years, *SD* = 2.42) from Vrije Universiteit Amsterdam with reported normal or corrected-to-normal visual acuity participated in Experiment 3. Participants were randomly assigned to either LS group or HS group so that each group ended up with 19 participants. Originally, we planned to have a sample size in each group matching with that in Experiments 1, and 2, but had to abort collecting more participants due to COVID-19.

The experimental setup was identical to Experiment 1, with the following exceptions: There were only two types of distractors in Experiment 3, a high-saliency size distractor with an increment of 1.0 d.v.a. in diameter (identical to the high-saliency distractor in Experiment 1), and a low-saliency size distractor with an increment of 0.65 d.v.a. in diameter (identical to the medium saliency distractor in Experiment 1). As in Experiment 1, each participant performed 12 experimental blocks of 180 trials each. Out of all the trials, one-sixth (i.e., 360 trials) were distractor-absent trials, while on the remaining trials (i.e., 1,800 trials) a low or high-saliency distractor would be presented. Both types of distractors were more likely (65% probability) to appear at one and the same location (the HPL) in the search display. Crucially, however, for the LS group, the low-saliency distractor appeared on 80% of all distractor-present trials, resulting in a total number of 1,440 low-saliency distractor trials and 360 high-saliency distractor trials; for the HS group, the high-saliency distractor appeared on 80% of all distractor-present trials, resulting in a total number of 1,440 high-saliency distractor trials and 360 low-saliency distractor trials. After performing the task, participants finished the same implicit learning questionnaire as in Experiments 1, and 2.

### Results

Trials on which the RTs were faster than 200 ms (1.3%) were excluded from the analyses.

#### Attentional capture

For both the LS and HS groups, we performed a one-way repeated-measures ANOVA on mean RTs and mean error rates, with distractor presence (absent vs. high-saliency distractor vs. low-saliency distractor) as a factor.

**Fig. 6:**
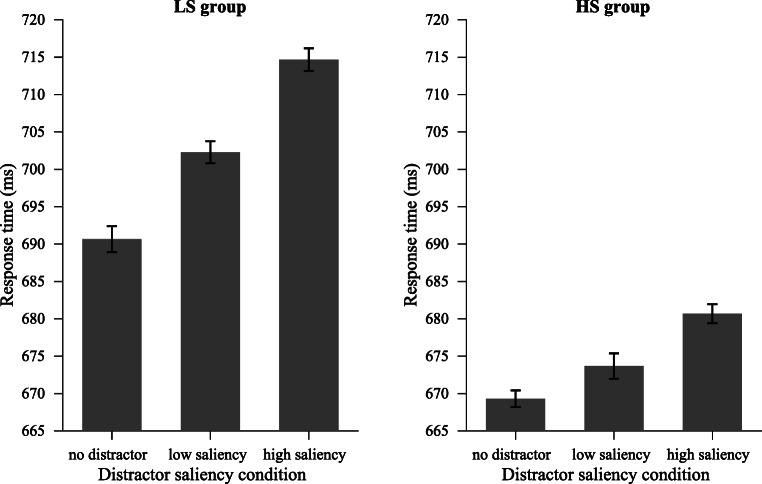
Experiment 3: Mean RTs for different distractor saliency conditions. Left panel: LS group (low-saliency distractors appeared on 80% of all distractor-present trials). Right panel: HS group (high-saliency distractors appeared on 80% of all distractor-present trials)

For the LS group, the effect of the distractor presence was significant, *F*(2, 36) = 38.65, *p* < .001, ${\eta}_p^2$= .68 (see Fig. 6, left panel). Planned comparisons showed that both low-saliency and high-saliency distractors interfered with target search, and the high-saliency distractor caused larger interference than the low-saliency distractor (absent vs. low-saliency distractor: 691 ms ± 72 vs. 702 ms ± 79), *t*(18) = 4.08, *p* < .001, *d* = 0.15; (absent vs. high-saliency distractor: 691 ms ± 72 vs. 715 ms ± 76), *t*(18) = 8.25, *p* < .001, *d* = 0.32; (low vs. high-saliency distractor), *t*(18) = 5.14, *p* < .001, *d* = 0.16. There was no evidence for a speed–accuracy trade-off (all error rate comparisons *p*s > .1 or congruent with RT effects).

For the HS group, there was also a main effect of distractor presence, *F*(2, 36) = 11.43, *p* < .001, ${\eta}_p^2$ = .39 (see Fig. 6, right panel). Planned comparisons also showed that the high-saliency distractor caused larger interference than the low-saliency distractor (low-saliency vs. high-saliency distractor: 674 ms ± 81 vs. 681 ms ± 82), *t*(18) = 2.51, *p* = .022, *d* = 0.08. There were no significant effects on error rates (*p*s > .1).

#### Saliency-specific suppression

To examine whether the saliency-specific pattern for both groups was consistent with Experiments 1, and 2, and whether the pattern differed between two groups, we performed a three-way mixed ANOVA on RT data, with distractor saliency (low vs. high) and distractor position (HPL vs. LPL) as within-subjects factors, and group (LS group vs. HS group) as a between-subjects factor. The results showed a strong three-way interaction between distractor saliency, distractor position, and group, *F*(1, 36) = 18.92, *p* < .001, ${\eta}_p^2$ = .34, indicating that there might be different effects for different groups, although the main effect of group seemed not significant, *F*(1, 36) = 1.58, *p* = .218. In the following, we examined the two-way effects for the LS group and HS group, respectively, to detail the possible differences.

**Fig. 7:**
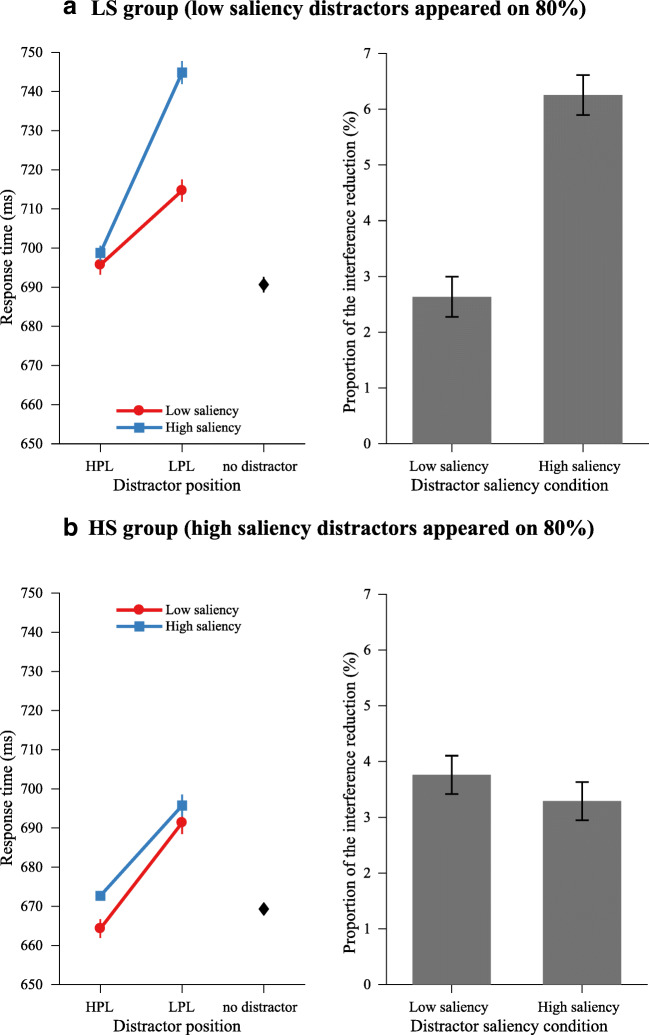
Experiment 3. Left panel: Mean RTs by distractor saliency over distractor position condition. Right panel: The proportion of reduction in interference (from the LPL to the HPL) for different distractor saliency conditions. Data from LS group and HS group are presented in subfigures (**a**) and (**b**), respectively

For the LS group, as shown in Fig. 7 (upper-left panel), the results showed a main effect of distractor saliency, *F*(1, 18) = 36.22, *p* < .001, ${\eta}_p^2$ = .67, and a main effect of distractor position, *F*(1, 18) = 97.96, *p* <. 001, ${\eta}_p^2$ = .85. Crucially, there was a strong interaction, *F*(1, 18) = 25.85, *p* < .001, ${\eta}_p^2$= .59, between these two factors. Subsequent comparisons showed that the magnitude of suppression was higher for the high-saliency distractor than the low-saliency distractor (46.1 ms ± 17.2 vs. 19.0 ms ± 19.6), *t*(18) = 5.08, *p* < .001, *d* = 1.43. The interference by the low-saliency distractor when it appeared at the HPL was reduced to an indistinguishable level from distractor-absent trials, *t*(18) = 1.44, *p* = .168, BF_01_ = 1.74, whereas RT when the high-saliency distractor appeared at the HPL was statistically longer than RT on distractor-absent trials, *t*(18) = 2.45, *p* = .025, *d* = 0.77. Analyses on error rate showed main effects on distractor saliency and distractor position (both congruent with RT effects), but no interaction (see Table 1 for mean RTs and error rates). Similar to Experiments 1, and 2, a *t* test on the proportion of the interference reduction showed that the interference of high-saliency distractors was reduced by a larger proportion than low-saliency distractors (low vs. high: 2.6% vs. 6.3%), *t*(18) = 5.02, *p* < .001, *d* = 1.38 (see Fig. 7, upper-right panel).

For the HS group, as shown in Fig. 7 (bottom-left panel), the results showed a main effect of distractor position, *F*(1, 18) = 89.79, *p* < .001, ${\eta}_p^2$ = .67, and a main effect of distractor saliency, *F*(1, 18) = 3.96, *p* = .062, marginally significant). However, the interaction between these two factors was not significant, *F*(1, 18) = 0.70, *p* = .413, BF_01_ = 2.70. Subsequent comparisons showed that the magnitude of suppression was indistinguishable for low-saliency and high-saliency distractors (27.0 ms ± 17.7 vs. 23.0 ms ± 13.0), *t*(18) = 0.84, *p* = .413, BF_01_ = 3.08. The mean RT when low-saliency and high-saliency distractors appeared at the HPL were basically equivalent to distractor-absent trials (absent vs. low-saliency distractor at the HPL), *t*(18) = 1.67, *p* = .113; (absent vs. high-saliency distractor at the HPL), *t*(18) = 1.91, *p* = .073. When comparing search performance when low-saliency and high-saliency distractors appeared at the HPL and LPL, we found that the mean RT when the high-saliency distractor appeared at the HPL was significantly longer than the low-saliency distractor, *t*(18) = 3.34, *p* = .004, while the mean RT at the LPL was statistically indistinguishable for low-saliency and high-saliency distractors, *t*(18) = 0.87, *p* = .398, BF_01_ = 3.02. Analyses on error rate only showed a main effect on distractor position (congruent with RT effect), but no effects on distractor saliency or interaction (see Table 1 for mean RTs and error rates). When comparing the proportion of the interference reduction, the results showed that the difference between low-saliency and high-saliency distractors was no longer reliable (low vs. high: 3.76% vs. 3.29%), *t*(18) = 0.69, *p* = .500 (see Fig. 7, bottom-right panel), which implies a suppression pattern that is different from the pattern in Experiment 1, 2 and LS group in Experiment 3.

#### Awareness assessment

Out of 38 participants, 16 indicated that they noticed certain regularities, while in the second question, only four correctly identified the HPL. After excluding these four participants from all analyses, all major findings remain the same (see Supplemental Materials).

### Discussion

In Experiment 3, our manipulation on the frequency of encountering distractors of different saliency levels led to different suppression patterns in the LS group compared with the HS group. For both groups, a rare distractor presented at a rare location (i.e., LPL) gave rise to strong capture, which is consistent with previous results that capture depends on the frequency of encountering a distractor (Geyer, Müller, & Krummenacher, 2008; Horstmann, 2002, 2005; Müller et al., 2009; Sayim, Grubert, Herzog, & Krummenacher, 2010). This frequency-induced strong capture for rare distractors presented at rare locations in turn reshaped the saliency-specific suppression pattern we observed in Experiments 1, and 2.

Specifically, for LS group, the saliency-specific suppression pattern was held and boosted (numerically, compared with that in Experiments 1, and 2): Strong capture by the rare high-saliency distractor at the LPL led to strong suppression of it, which resulted in a down-weighting factor (i.e., the proportion of the interference reduction) for the rare high-saliency distractor nearly twice as large as that of the frequent, low-saliency distractor (see Fig. 7, upper-right panel). While for the HS group, the saliency-specific suppression pattern breaks down and even reversed: The infrequent, low-saliency distractor caused an unusually large interference at the LPL; as a result, the down-weighting factor of the rare low-saliency distractor was even numerically larger than that of the frequent, high-saliency distractor, though statistically insignificant (see Fig. 7, bottom-right panel).

## General discussion

The current study provides evidence for a saliency-specific mechanism of distractor suppression that has dimension-independent aspects. Experiment 1 demonstrated that when distractors of different saliency levels had the same HPL, the more salient a distractor, the more suppression was applied when it appeared at the HPL. This was observed within size dimension, which has not been tested before. Therefore, our results also extend the generality of distractor suppression, and confirms the importance of learning to suppress a distractor in attentional control. Experiment 2 also showed saliency-specific suppression, and because low-saliency and high-saliency distractors were defined on color and size dimensions, respectively, we established that this saliency-specific suppression was not confined within a single feature dimension, but instead can extend to different feature dimensions.

Consistent with previous findings (Wang & Theeuwes, 2018a, 2018b, 2018c), attentional capture was reduced when distractors were presented at the HPL relative to LPLs. In addition and consistent with previous studies, in all experiments, we showed that in the no-distractor condition the selection of the target was less efficient (longer RTs) when it was presented at the HPL relative to when it appeared at the LPL (see Supplemental Materials). These findings are very much in line with a pure location-based suppression account, in which it is assumed that before display onset, the location that is most likely to contain a distractor is proactively suppressed (Theeuwes, 2019). For example, Wang, van Driel, Ort, and Theeuwes (2019) demonstrated proactive suppression using EEG showing enhanced alpha power contralateral to the HPL about 1,000 ms before display onset. In addition to prestimulus enhanced alpha power, there was early PD component (80 ms poststimulus), signifying early suppression. However, in the current study in which distractors had different saliency levels and were all presented at the same HPL, if there would have been location-based suppression only, the magnitude of suppression would have been the same for different types of distractors, which is not in line with our results. We therefore assume that a pure location-based suppression cannot explain all the results; instead we assume that in addition to proactive location-based suppression there is also a saliency-specific suppression component. Specifically, we argue for a saliency-specific suppression that modulates suppression on a trial-by-trial basis, contingent on the saliency of the actual distractor presented.

Experiment 3 demonstrates that by manipulating the frequency of presence for distractors of different saliency levels, saliency-specific suppression is modulated. In the condition where an infrequent, low-saliency distractor appears at the LPL, it will cause an unusually large interference. As a result, there will be a higher proportion of interference reduction (from the LPL to the HPL) for the low-saliency distractor, which in turn breaks down the saliency-specific suppression pattern. The results from Experiment 3 show a high level of plasticity within the spatial priority map allowing to optimize behavior in a specific context (Chelazzi et al., 2014; Geng, 2014).

The current findings showing saliency-specific suppression should be compared with another study using the additional singleton paradigm in which there was one HPL that contained either a red or a green color distractor presented among grey elements (Wang & Theeuwes, 2018c, Experiment 4). Across participants, one color of the distractor was presented much more often than the other color. For example, one group of participants encountered in 80% of trials a red color singleton distractor, while in the remaining 20% of trials a green color distractor singleton (and vice versa for the other group). Critically for the present discussion, the green and red color distractor were about equal in saliency. If the system would adapt to the frequency of the distractor color encountered, one would expect that there would be differences in the amount of suppression when the frequent color distractor would be presented at the HPL relative to when the infrequent color would be presented at that location. Critically, there was no effect whatsoever of the frequency of the color feature of the distractor: suppression was just as strong for the frequent color as for the infrequent color, providing strong evidence that statistical learning about specific features does no play a role in suppression. The current findings are consistent with this notion and show that it is not the specific feature that plays a role but instead the saliency encountered. From a functional point of view, it would make much more sense if suppression is adaptive to the saliency encountered as the saliency determines the amount of distraction a distractor can cause.

Overall, the current findings are inconsistent with the recent study of Gaspelin and Luck (2018), who claimed that first-order feature information is needed to suppress distractors. In other words, they claimed that suppression can be achieved only when there is foreknowledge of the upcoming distractor’s feature value (e.g., red, vertical). Our study shows strong evidence for a global saliency suppression account, according to which the visual system can suppress a salient distractor irrespective of feature dimensions that drive this saliency, and suppression can be applied without foreknowledge of the upcoming distractor’s feature value (which is varied randomly across all trials in the current study).

Notably, our analysis also shows that an alternative explanation that suppression simply reduces the impact of any distractor by a certain proportion of the initial interference is not tenable. Instead, the amount of suppression when a distractor appears at the HPL is contingent on the saliency of each distractor, respectively. However, there might be concerns that ruling out this possibility by calculating normalized interference reduction effects on RTs (i.e., proportion of the interference reduction) seems to be somewhat oversimplified. To examine into how statistical learning alters the distractor activations within the spatial priority map and further impacts the observed RTs, more sophisticated approaches like mathematical modelling might be needed in future research on this topic (for further discussion, see Liesefeld & Müller, in press).

In view of the position we adhere here that suppression has both location-based and saliency-specific components, it is feasible that the recent finding from Failing and Theeuwes (2020) can also be interpreted as a combination of these two factors. In their study, there were two HPL locations, which were more likely to contain a high-saliency distractor and a low-saliency distractor, respectively. The results in their study showed that there was more suppression for the HPL of the high-saliency distractor; yet in their study it was difficult to explain why an infrequent, low-saliency distractor was not more suppressed when it was presented at the HPL of the high-saliency distractor. If one assumes that there is location-based suppression when an infrequent distractor appears at an HPL and a combination of both saliency-based and location-based suppression when the frequent distractor is presented at the HPL, one is able to explain why a low-saliency distractor, when presented at the location that is more likely to contain a high-saliency distractor, is not more suppressed. Future studies should shed some light on this.

Many studies on the neural mechanisms of distractor suppression have focused on the role of higher-level cortical areas (DiQuattro & Geng, 2011; Kane & Engle, 2002; Shimamura, 2000) in suppressing distractors, while there has also been evidence showing that distractor suppression is implemented through modulations within visual cortex (Gazzaley et al., 2007; Seidl, Peelen, & Kastner, 2012). In the present study, we take the latter view and get insights from the V1 saliency theory (Li, 2002; Zhang, Zhaoping, Zhou, & Fang, 2012; Zhaoping, 2008; Zhaoping & May, 2007) to interpret the possible neural mechanisms of saliency-specific suppression. Specifically, the saliency of an item can be represented by the highest neural response among all the V1 cells, and our findings of a saliency-specific suppression can be explained as the neural adaptation of V1 cells that cover the HPL with their classical receptive fields. The neural adaptation is tuned in accordance to the firing rates of the group of V1 cells representing the distractor, which is finally reflected on the saliency-specific reduction of interference when the distractor appears at the HPL. This novel interpretation has the advantage of explaining why participants were generally unaware of the location that was more likely to contain a distractor. That is, if suppression can be implemented by modulating the neural representation in V1, it means that attentional suppression can occur before we are aware of the actual feature and location of the distractor, because the features and locations that V1 cells encode can only be made aware of by the relay of neural signals to higher-cortical areas. The most important differences between V1 saliency theory and traditional saliency models is that the former claims that no separate feature maps or subsequent combinations of them are needed to generate the saliency map.

In summary, the present study provides evidence for a saliency-specific mechanism of distractor suppression that is independent of feature dimensions. We argue that this attentional bias is acquired through statistical learning, which in turn allows for a highly flexible, saliency-dependent adaption modulating suppression on a trial-by-trial basis, contingent on the saliency encountered.

## Electronic supplementary material

ESM 1 (DOCX 74 kb)
