# Supplementary material for: A saliency-specific and dimension-independent mechanism of distractor suppression
Source: Atten Percept Psychophys. 2020 Oct 6;83(1):292–307. doi: 10.3758/s13414-020-02142-8 (PMC7538281; doi:10.3758/s13414-020-02142-8)
Supplement: Supplementary file 1 — (DOCX 74 kb) [file 13414_2020_2142_MOESM1_ESM.docx]

**Supplemental Materials**

**Exp. 1** Additional Results

**Location-based suppression.** To examine the effect of location-based suppression that has been observed in previous studies, a repeated measures ANOVA on RT data with distractor location (absent vs. HPL vs. LPL) as factor was performed. As shown in **Fig. S1** (left panel), the results showed a significant main effect (*F*(2,60) = 51.28, *p* < .001, $\eta_{p}^{2}$ = .63). Subsequent comparisons revealed that both HPL and LPL interfered significantly with target search (absent vs. HPL, *t*(30) = 4.57, *p* < .001, *d* = .13; absent vs. LPL, *t*(30) = 7.93, *p* < .001, *d* = .39). Consistent with Wang and Theeuwes (2018a, 2018b, 2018c), when the distractor appeared at the HPL it interfered less compared to when it appeared at an LPL (*t*(30) = 6.88, *p* < .001, *d* = .26). There were no significant differences in error rate (*p*s > .1), which excludes the alternative explanation of a speed-accuracy trade-off. In the next step, we also examined the RTs and error rates on distractor-absent trials to further confirm the effect of location-based suppression. As shown in **Fig. S1** (right panel), the results showed that participants were slower when the target was presented at the HPL relative to when it was presented at the LPL (*t*(30) = 2.85, *p* = .008, *d* = .26), which is consistent with previous studies (Wang & Theeuwes, 2018a, 2018b, 2018c). There was no effect on error rates, *t*(30) = .29, *p* = .776. These findings demonstrate that target search was impaired even when the distractor was absent, thus further confirming the effect of location-based suppression.

**Fig. S1** Experiment 1: **Left panel:** mean RTs for different distractor location conditions. **Right panel:** mean RTs when the target is presented at the HPL and LPL location in the no-distractor condition.

**Intertrial location priming.** To rule out the possibility that suppression can be explained by intertrial location priming (Maljkovic & Nakayama, 1994), we excluded trials in which the position of the distractor on the current trial was identical to the previous trial and submitted RTs of the remaining trials to an ANOVA with distractor saliency (low vs. medium vs. high) and distractor position (HPL vs. LPL) as factors. The results replicated all major findings. There were a main effect of distractor saliency (*F*(2,60) = 45.57, *p* <.001, $\eta_{p}^{2}$= .60), a main effect of distractor position (*F*(1,30) = 48.11, *p* < .001, $\eta_{p}^{2}$ = .62), as well as interaction (*F*(2,60) = 20.19, *p* < .001, $\eta_{p}^{2}$ = .40). Individual comparisons showed that the saliency-specific suppression pattern remained robust after removing all trials with repeated distractor position. The amount of suppression (difference between HPL and LPL) increases with the saliency of the distractor: for low saliency the amount of suppression is *M* = 4.9 ms ± 22.4, for medium saliency 22.6 ms ± 21.4 and for high saliency it is 39.1 ms ± 29.9. All these values differed from each other significantly (*p*s < .01). There was no evidence for a speed-accuracy trade-off (all error rate comparisons *p*s > .1 or congruent with RT effects). These demonstrate that the saliency-specific suppression effects cannot be explained by intertrial location priming.

**Fig. S2** Experiment 1: Mean RTs by distractor saliency for different distractor positions relative to the HPL. Dist-0 represents exactly the HPL, dist-1 the location next to the HPL, dist-2 two locations away, dist-3 three locations away from the HPL, and dist-4 is the location opposite to the HPL. Data are collapsed across conditions in which the distractor was presented at symmetric positions along the vertical meridian.

**Spatial gradient of suppression.** We further investigated the spatial distribution of the suppression effect. If the saliency-specific suppression has a spatial extent, then when the distractor appeared farther away from the HPL, we would expect to see a steeper rise of RTs for distractors with a higher saliency. According to the distance of different locations to the HPL, we divided the distractor locations into five categories, ranging from distance 0, which means exactly the HPL, to distance 4, which means the location that was opposite to the HPL. As shown in **Fig. S2**, a repeated measures ANOVA with distractor saliency (low vs. medium vs. high) and the distance of the distractor to the HPL (distance 0 to distance 4) as factors showed a main effect of distractor saliency (*F*(2,60) = 53.10, *p* < .001, $\eta_{p}^{2}$ = .64) and distance (*F*(4,120) = 15.55, *p* < .001, $\eta_{p}^{2}$ = .34) as well as a significant interaction (*F*(8,240) = 2.70, *p* = .007, $\eta_{p}^{2}$ = .08). There was no evidence for a speed- accuracy trade-off (all error rate comparisons *p*s > .1 or congruent with RT effects). These demonstrate a saliency-specific spatial gradient of suppression, offering further supporting evidence for a saliency-specific suppression.

**Awareness assessment.** After excluding two participants who correctly identified the HPL, we submitted RTs of the remaining trials to an ANOVA with distractor saliency (low vs. medium vs. high) and distractor position (HPL vs. LPL) as factors. The results replicated the major findings. There were a main effect of distractor saliency (*F*(2,56) = 51.08, *p* <.001, $\eta_{p}^{2}$ = .65), a main effect of distractor position (*F*(1,28) = 49.12, *p* < .001, $\eta_{p}^{2}$ = .64), as well as interaction (*F*(2,56) = 26.61, *p* < .001, $\eta_{p}^{2}$ = .49).

**Exp. 2** Additional Results

**Location-based suppression.** To investigate the effect of location-based suppression, an ANOVA on RT with distractor location (absent vs. HPL vs. LPL) as factor was performed. As shown in **Fig. S3** (left panel), the results showed a main effect on distractor location (*F*(2,62) = 57.46, *p* < .001, $\eta_{p}^{2}$= .65). Subsequent comparisons showed that both HPL and

**Fig. S3** Experiment 2. **Left panel:** mean RTs for different distractor location conditions. **Right panel:** mean RTs when the target is presented at the HPL and LPL location in the no-distractor condition.

LPL interfered significantly with target search (absent vs. HPL, *t*(31) = 2.35, *p* = .025, *d*= .07; absent vs. LPL, *t*(31) = 8.21, *p* < .001, *d* = .38). As expected, when the distractor appeared at the HPL it interfered less relative to when it appeared at an LPL (*t*(31) = 9.36, *p* < .001, *d* = .31). No significant differences were revealed in error rates, *F*(2,62) = 2.22, *p* = .118. To further confirm the effect of location-based suppression, we examined the RTs and error rates on distractor-absent trials. As shown in **Fig. S3** (right panel), the results showed that participants were slower when the target was presented at the HPL relative to when it was presented at the LPL, *t*(31) = 2.39, *p* = .023, *d* = .27. There was no effect on error rates, *t*(31) = .30, *p* = .764. Consistent with previous studies (Wang & Theeuwes, 2018a, 2018b, 2018c), these findings substantiate that target selection was less efficient when the target was presented at the HPL relative to LPLs, signifying the effect of location-based suppression.

**Intertrial location priming.** To assess whether suppression can be explained by intertrial location priming, we excluded trials in which the position of the distractor repeated between trials. We then submitted RTs of the remaining trials to an ANOVA with distractor saliency (low vs. high) and distractor position (HPL vs. LPL) as factors. The results replicated the major findings. There were a main effect of distractor saliency (*F*(1,31) = 24.94, *p* < .001, $\eta_{p}^{2}$ = .45), a main effect of distractor position (*F*(1,31) = 60.67, *p* < .001, $\eta_{p}^{2}$ = .66), as well as interaction (*F*(1,31) = 7.92, *p* = .038, $\eta_{p}^{2}$= .25). Again, these findings demonstrate that the saliency-specific suppression effects cannot be explained by intertrial location priming.

**Spatial gradient of suppression.** We also determined the spatial distribution of the suppression effect. If the saliency-specific suppression extends spatially, then when the distractor appeared farther away from the HPL, there should be a steeper rise of RTs for the high saliency size distractor relative to the low saliency color distractor. As shown in **Fig. S4**, a repeated measures ANOVA with distractor saliency (low vs. high) and the distance of the distractor to the HPL (distance 0 to distance 4) as factors showed a main effect of distractor saliency (*F*(1,31) = 24.86, *p* < .001, $\eta_{p}^{2}$ = .45) and distance (*F*(4,124) = 9.73, *p* < .001, $\eta_{p}^{2}$ = .24), as well as a significant interaction (*F*(4,124) = 2.66, *p* = .036, $\eta_{p}^{2}$ = .08).

**Fig. S4** Experiment 2: Mean RTs by distractor saliency for different distractor positions relative to the HPL.

No significant effects on error rate were found. These demonstrate a saliency-specific spatial gradient of suppression which is similar to the pattern we observed in Experiment 1, thus supporting our claim of a saliency-specific suppression.

**Awareness assessment.** After excluding five participants who correctly identified the HPL, we submitted RTs of the remaining trials to an ANOVA with distractor saliency (low vs. high) and distractor position (HPL vs. LPL) as factors. The results replicated the major findings. There were a main effect of distractor saliency (*F*(1,26) = 25.46, *p* <.001, $\eta_{p}^{2}$ = .50), a main effect of distractor position (*F*(1,26) = 80.11, *p* < .001, $\eta_{p}^{2}$ = .76), as well as interaction (*F*(1,26) = 9.10, *p* = .006, $\eta_{p}^{2}$ = .26).

**Exp. 3** Additional Results

**Location-based suppression.** For both LS and HS groups, we submitted RT data to an ANOVA with distractor location (absent vs. HPL vs. LPL) as factor.

For LS group, the results showed a main effect on distractor location (*F*(2,36) = 42.66, *p* < .001, $\eta_{p}^{2}$= .70; see **Fig. S5**, upper-left panel). Subsequent comparisons showed that the LPL interfered significantly with target search (*t*(18) = 9.53, *p* < .001, *d* = .39), and the HPL interfered less relative to the LPL (*t*(18) = 6.32, *p* < .001, *d* = .30). The interference when distractors appeared at the HPL was so far reduced that the search performance was indistinguishable from distractor-absent trials (*t*(18) = 1.70, *p* = .106, BF_01_ = 1.25). The effects on error rate were congruent with RT effects. To further confirm the effect of location-based suppression, we examined the RTs and error rates on distractor-absent trials. As shown in **Fig. S5** (upper-right panel), the results showed that participants were slower when the target was presented at the HPL relative to when it was presented at the LPL, *t*(18) = 2.31, *p* = .033, *d* = .37. There was no effect on error rates, *t*(18) = .75, *p* = .46. This means that target search was impaired on distractor-absent trials, further confirming the effect of location-based suppression.

For HS group, the results also showed a main effect on distractor location (*F*(2,36) = 81.62, *p* < .001, $\eta_{p}^{2}$= .82; see **Fig. S5**, bottom-left panel). Subsequent comparisons showed that the LPL interfered significantly with target search (*t*(18) = 11.44, *p* < .001, *d* = .30), and the HPL interfered less relative to the LPL (*t*(18) = 9.22, *p* < .001, *d* = .28).

1. **LS group (low saliency distractors appeared on 80%)**

1. **HS group (high saliency distractors appeared on 80%)**

**Fig. S5** Experiment 3. **Left panel:** mean RTs for different distractor location conditions. **Right panel:** mean RTs in the no-distractor condition. Data from LS group and HS group are presented in subfigures (**A**) and (**B**), respectively.

Similar to LS group, the interference when distractors appeared at the HPL was so far reduced that the search performance was indistinguishable from distractor-absent trials (*t*(18) = .93, *p* = .363, BF_01_ = 2.87). The effects on error rate were also congruent with RT effects. We then examined the RTs and error rates on distractor-absent trials. As shown in **Fig. S5** (bottom-right panel), the results showed that participants were slower when the target was presented at the HPL relative to when it was presented at the LPL, *t*(18) = 2.06, *p* = .054, marginally significant. There was no effect on error rates, *t*(18) = 1.65, *p* = .116. These demonstrate that, for HS group, we also found the effect of location-based suppression.

**Intertrial location priming.** For both LS and HS groups, we excluded trials in which the position of the distractor repeated between trials, and then submitted RTs of the remaining trials to an ANOVA with distractor saliency (low vs. high) and distractor position (HPL vs. LPL) as factors. For both groups, the results replicated the major findings. For LS group, the main effects of distractor saliency (*F*(1,18) =17.92, *p* <.001, $\eta_{p}^{2}$ = .50 and distractor position (*F*(1,18) = 72.99, *p* <.001, $\eta_{p}^{2}$ = .80), as well as interaction (*F*(1,18) = 30.70, *p* < .001, $\eta_{p}^{2}$ = .63) were significant. For HS group, the main effect of distractor position was significant (*F*(1,18) = 64.50, *p* <.001, $\eta_{p}^{2}$ = .78). These demonstrate that the results cannot be explained by intertrial location priming.

**Spatial gradient of suppression.** We also investigated the spatial gradient of suppression for both groups by performing a repeated measures ANOVA with distractor saliency (low vs. high) and the distance of the distractor to the HPL (distance 0 to distance 4) as factors.

**Fig. S6** Experiment 3: Mean RTs by distractor saliency over distractor position. **Left panel:** LS group (low saliency distractors appeared on 80%). **Right panel:** HS group (low saliency distractors appeared on 80%).

For LS group, as shown in **Fig. S6** (left panel), the results showed a main effect of distractor saliency (*F*(1,18) = 45.13, *p* < .001, $\eta_{p}^{2}$ = .72) and distance (*F*(4,72) = 10.18, *p* < .001, $\eta_{p}^{2}$ = .36) as well as a significant interaction (*F*(4,72) = 3.28, *p* = .016, $\eta_{p}^{2}$ = .15). Analyses on error rate only found a significant effect on distractor saliency (congruent with RT effect).

For HS group, as shown in **Fig. S6** (right panel), a main effect on distance was found (*F*(4,72) = 8.76, *p* < .001, $\eta_{p}^{2}$ = .33), but there were no effects on distractor saliency (*F*(1,18) = 1.97, *p* = .177, BF_01_ = 29918.16) or interaction (*F*(4,72) =.75, *p* = .562, BF_01_ = 9.97). Similar analyses on error rate only found a significant effect on distractor saliency (congruent with RT effect).

**Awareness assessment.** We excluded four participants in total who correctly identified the HPL for LS and HS groups. For LS group, after excluding two participants, there was still a main effect of distractor saliency(*F*(1,16) = 28.94, *p* <.001, $\eta_{p}^{2}$ = .64), a main effect of distractor position (*F*(1,16) = 81.61, *p* < .001, $\eta_{p}^{2}$ = .84), as well as interaction (*F*(1,16) = 19.57, *p* < .001, $\eta_{p}^{2}$= .55). For HS group, after excluding two participants, the major findings were also replicated: the results showed a main effect of a main effect of distractor position (*F*(1,16) = 97.70, *p* < .001, $\eta_{p}^{2}$ = .86) and a main effect of distractor saliency(*F*(1,16) = 4.38, *p* = .053, marginally significant), but no interaction between these two factors (*F*(1,16) = 0.52, *p* = .482, BF_01_ = 11.74).

References:

Maljkovic, V., & Nakayama, K. (1994). Priming of pop-out: I. Role of features. *Memory & Cognition, 22*(6), 657-672. doi:10.3758/bf03209251

Wang, B., & Theeuwes, J. (2018a). How to inhibit a distractor location? Statistical learning versus active, top-down suppression. *Attention Perception & Psychophysics, 80*(4), 860-870. doi:10.3758/s13414-018-1493-z

Wang, B., & Theeuwes, J. (2018b). Statistical regularities modulate attentional capture. *J Exp Psychol Hum Percept Perform, 44*(1), 13-17. doi:10.1037/xhp0000472

Wang, B., & Theeuwes, J. (2018c). Statistical regularities modulate attentional capture independent of search strategy. *Attention Perception & Psychophysics, 80*(7), 1763-1774. doi:10.3758/s13414-018-1562-3
